# Implications of differentiated care for successful ART scale-up in a concentrated HIV epidemic in Yangon, Myanmar

**DOI:** 10.7448/IAS.20.5.21644

**Published:** 2017-07-21

**Authors:** Anita Mesic, Julie Fontaine, Theingy Aye, Jane Greig, Thin Thin Thwe, Laura Moretó-Planas, Jarmila Kliesckova, Khin Khin, Nana Zarkua, Lucia Gonzalez, Erwin Lloyd Guillergan, Daniel P. O’Brien

**Affiliations:** ^a^ Médecins Sans Frontières, Operational Center Amsterdam, The Netherlands; ^b^ Médecins Sans Frontières, Operational Center Amsterdam, Yangon, Myanmar; ^c^ Médecins Sans Frontières, Operational Center Amsterdam, UK, London

**Keywords:** HIV, Myanmar, differentiated care, task shifting, integration

## Abstract

**Introduction**: National AIDS Programme in Myanmar has made significant progress in scaling up antiretroviral treatment (ART) services and recognizes the importance of differentiated care for people living with HIV. Indeed, long centred around the hospital and reliant on physicians, the country’s HIV response is undergoing a process of successful decentralization with HIV care increasingly being integrated into other health services as part of a systematic effort to expand access to HIV treatment. This study describes implementation of differentiated care in Médecins Sans Frontières (MSF)-supported programmes and reports its outcomes.

**Methods**: A descriptive cohort analysis of adult patients on antiretroviral treatment was performed. We assessed stability of patients as of 31 December 2014 and introduced an intervention of reduced frequency of physicians’ consultations for stable patients, and fast tract ART refills. We measured a number of saved physician’s visits as the result of this intervention. Main outcomes, remained under care, death, lost to follow up, treatment failure, were assessed on 31 December 2015 and reported as rates for different stable groups.

**Results**: On 31 December 2014, our programme counted 16, 272 adult patients enrolled in HIV care, of whom 80.34% were stable. The model allowed for an increase in the average number of patients one medical team could care for – from 745 patients in 2011 to 1, 627 in 2014 – and, thus, a reduction in the number of teams needed. An assessment of stable patients enrolled on ART one year after the implementation of the new model revealed excellent outcomes, aggregated for stable patients as 98.7% remaining in care, 0.4% dead, 0.8% lost to follow-up, 0.8% clinical treatment failure and 5.8% with immunological treatment failure.

**Conclusions**: Implementation of a differentiated model reduced the number of visits between stable clients and physicians, reduced the medical resources required for treatment and enabled integrated treatment of the main co-morbidities. We hope that these findings will encourage other stakeholders to implement innovative models of HIV care in Myanmar, further expediting the scale up of ART services, the decentralization of treatment and the integration of care for the main HIV co-morbidities in this context.

## Introduction

While Myanmar’s national HIV prevalence is below 1% there were 224, 794 people living with HIV in Myanmar in 2015 [1,2]. The epidemic is concentrated in urban areas and among key populations. HIV prevalence is estimated at 28.5% among people who inject drugs (PWID), 14.6% among female sex workers (FSW), and 11.6% among men who have sex with men (MSM) [2]. The National AIDS Programme (NAP) and partner organizations have made important gains in enhancing Myanmar’s HIV response; since 2015, access to HIV services has been available through 124 ART initiation sites, 173 decentralized sites set up to facilitate follow-up care for patients already on ART as well as 50 sites run by private-sector providers. As a result, HIV treatment coverage has doubled since 2012, reaching 57% of the affected population in 2016, while new HIV infections and HIV-related deaths have gone down [1]. Having long employed an HIV treatment model in which care is predominantly provided by specialists physicians in hospitals, Myanmar is now transitioning to a decentralized response that integrates HIV care into existing health services and improves access to treatment for the most prevalent co-morbidities. Still, despite these achievements, HIV treatment gap in Myanmar remains [1].

Myanmar bears a significant burden of HIV-associated TB, with incidence rate of 32 per 100, 000 in 2015 [3]. Yet in 2015, only 38% of patients co-infected with HIV and TB were enrolled on ART [3]. The National Tuberculosis Programme (NTP) and NAP recognize the importance of a joint response, and scaling up integrated HIV-TB activities is regarded as a priority. An additional threat to HIV key affected populations in Myanmar is co-infection with hepatitis B and C, with prevalence rates of HIV-HCV co-infection as high as 80% among PWID [4–6]. Moreover, with socio-economic development in Myanmar, there is an increased concern about non-communicable diseases (NCDs). While there are no published data on the prevalence of NCDs among HIV-infected populations in Myanmar per se, the National STEPS Survey (2014) reported a high prevalence of NCD risk factors, with almost all survey participants reporting at least one risk factor for NCDs. Some 26% of survey participants reported having hypertension while 10% reported having diabetes [7].

With a population of 7 million, Yangon is Myanmar’s largest city with some of the highest prevalence rates of HIV and TB infection in the country, particularly among the key populations [2,8]. The MSF project currently operates through two clinics in Yangon, where it has provided comprehensive HIV and TB care since 2003. Before the NAP initiated systematic scale up of ART services in 2010, MSF was the largest ART provider in the city, reaching more than 17,000 patients. Since 2014, MSF has restricted enrolment to children and adolescents and to adult patients with criteria of advance HIV infection, treatment failure, membership in a key population group, and co-morbidities; newly enrolled stable adult patients receive care through the National AIDS Programme. During the prior period of active general enrolment (2003–2014), MSF’s clinics were overwhelmed with new patients presenting with late-stage disease, with the median CD4 at enrolment being 71 cells/mm^3^ [9]. Adding to that challenge was the urgent health threat posed by increasing rates of drug-resistant tuberculosis, and in 2009, MSF partnered with the NTP to launch the pilot Drug Resistant TB (DRTB) Programme. Meanwhile, the aging MSF HIV cohort was afflicted with a growing burden of hypertension and diabetes, demanding integration of additional elements of chronic care.

Nearing the limits of its operational capacity to care for the large cohort on ART and an increasing number of severely sick new patients, MSF began investigating innovative approaches to service delivery. The MSF facility-based model of care was designed to optimize HIV care by differentiating ART delivery according to age and clinical characteristics, thereby allowing programs to save specialized expertise for the management of complicated cases with severe co-morbidities. The model was based on existing evidence for the facility based differentiated models showing good treatment outcomes with improved adherence, increasing patient and staff satisfaction, reduction of waiting time and service delivery for more patients with the same number of staff [10–12].

WHO 2016 Guidelines recommend differentiated HIV care as a key to scaling up ART programming, together with task shifting and decentralization [13]. As emphasized by the recently endorsed National Strategic Plan on HIV and AIDS 2016–2020, the complex epidemiological profile of HIV in Myanmar demands an innovative response that can more efficiently and effectively utilize limited resources [1,14]. Our report on differentiated HIV care in Myanmar, aims to outline the principles that guide this patient-centred approach and its outcomes to date, providing stakeholders in Myanmar with a blueprint for reaching the 90–90–90 targets.

## Methods

### Ethics review

We have used only anonymized secondary data and no intervention or patient contact was made for research purposes, therefore the issue of informed consent did not apply. The study met the criteria for analysis of routinely collected program data of the MSF independent Ethics Review Board [15] and the exemption from ethics review was granted by The National AIDS Programme Myanmar.

### Study population

The study population included all adult patients (age ≥18 years) on ART at MSF Clinics in Yangon on 31 December 2014, based on differentiation of clinical condition applying for adult patients only. The outcome analysis was performed for stable patients assigned to group B or C. Table 1 provides definition and criteria used for stability assessment and specific of the groups.

**Table 1.** Summary of the new and past service delivery model.

	New service delivery model			Past service delivery model
	A = unstable^a^	B = stable, short-term	C = stable, long-term	No differentiation to stability groups
Criteria	Adults <6 months of ART, or Patients with: Opportunistic infection (e.g. DRTB) Drug toxicity Diabetes or hypertension until condition is stabilized on adequate treatment Other medical condition requiring closer follow up Patients with adherence problems Patients changing ART regimen Children and adolescents	Adults ≥6 months and <12 months on ART with good clinical and immunological response and adherence to treatment of HIV and other relevant medical conditions Stable patients with diabetes mellitus and/or hypertension Stable second-line ART patients	Patients on ART ≥12 months with good clinical and immunological response to ART	All patients
WHO? Clinical consultation	Physician each time	Physician/nurse alternate	Nurse Physician by referral from the nurse	Physician
WHO? ART refill	Not eligible for direct ART refills	Not eligible for direct ART refills	Pharmacist/Dispenser	Direct ART refills not implemented. All ART refills done after the consultation by a physician and all patients receive therapeutic education by a nurse on each visit
WHEN? Clinical consultation and/or ART refill	Depending on the clinical and psychosocial condition	3 monthly	6 monthly3 monthly ART refill	1–2 monthly, depending on the duration of ART, reported adherence and clinical condition
WHEN? Counselling	Each visit first 6 months then if referral from physician/nurse or self referral	If referral from physician/nurse or self referral	If referral from physician/nurse or self referral	Each visit

^a^At this stage of the implementation, children and adolescents (age ≤18 years) are still not assessed for stability group and are followed up as group A.

### Model of care

**Figure 1.**
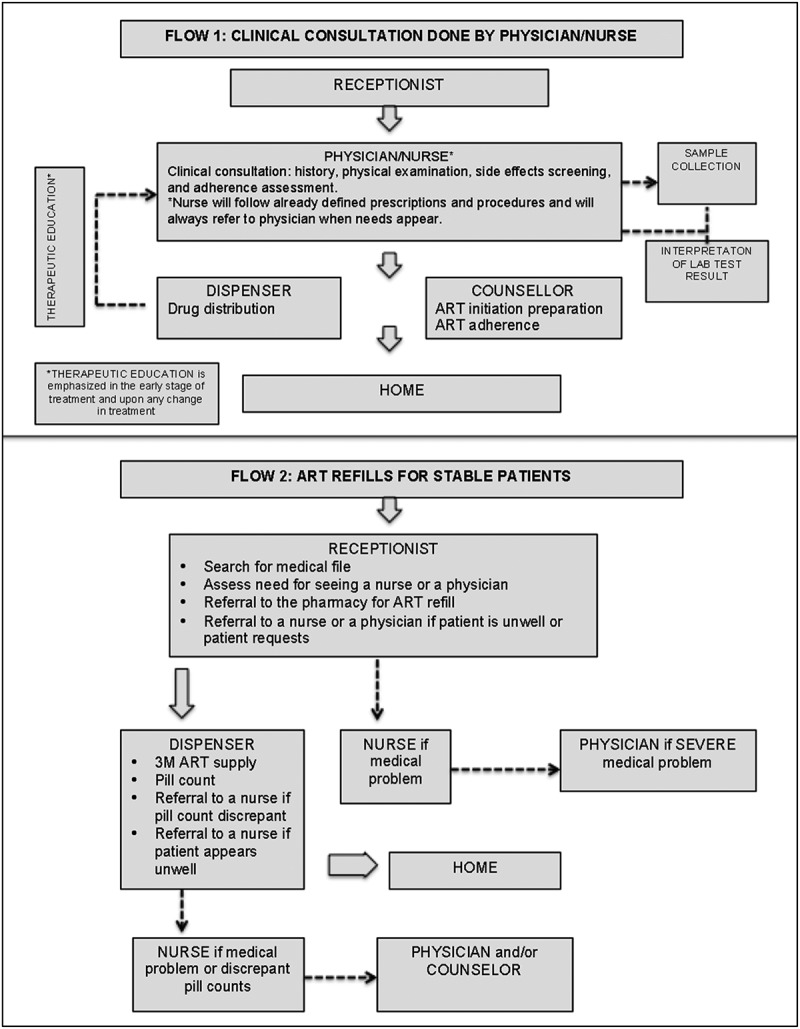
Patient flow. All patients report to the reception first and according to the instructions written in the medical file patients are referred to consultation by a physician or a nurse (Flow 1) or directly to the pharmacy together with prewritten prescription saved in the medical file (Flow 2).

HIV care in MSF Clinics in Yangon is provided by medical teams consisting of a physician, a nurse, and a counsellor. In 2010, MSF initiated the stepwise implementation of a new model based on three principles: Having on hand a three-month supply of drugs (up from one month); differentiation of adult patients according to their clinical condition, divorcing clinical consultations from ART refills; and shifting of clinical consultations for stable patients from physicians to nurses. With regards to the latter, the nurse does not make clinical decisions, except to follow already defined prescriptions and procedures (e.g. laboratory tests), and will refer to the physician as needed [14,16]. Stable patients are defined as belonging to one of 3 groups according to clinical stability, time on ART, treatment tolerance and adherence, and the presence of co-morbidities (Table 1). The stability group (designated A, B, C) is assigned by the physician. New adult patients who are starting ART remain in group A (unstable) and are consulted by the physician on each visit, with a frequency determined by their clinical condition. After 6 months on ART, the patient’s first clinical and immunological assessment is performed by a physician, while his or her psychosocial condition is assessed by a counsellor. Depending on the patient’s clinical and immunological status and adherence to treatment, the patient may be moved to group B, with scheduled appointments every three months alternating between a nurse and physician. Patients with chronic co-morbidities (diabetes, hypertension) will remain in group B. A second assessment is performed by a physician at 12 months. Clinically and immunologically stable patients enter group C, whereby, independently of clinical consultations done by a nurse every six months, ART refills are provided directly in the pharmacy (Figure 1).

Referral criteria from nurses to physician, including any clinical deterioration, as well as the detection of treatment failure defined by the development of WHO stage 3 and 4 co-morbidity, or a drop in CD4 count by 30% or presence of side effects of the treatment. In order to better address the needs of HIV-positive children and adolescents, certain days every week are set aside for their care. The objectives of this model are to improve patient flow (Figure 1) and free up physician time and space for the sickest patients, a group mainly comprised of patients presenting with advance HIV disease, failing ART, and those co-infected with DR-TB.

## Analysis

Routinely collected patient-level data entered in the FUCHIA software system (version 1.7.1 Epicentre, France) were used. Descriptive analysis was performed using STATA (version 14, StataCorp, Unites States of America). Patients’ characteristics comprised gender, age, time on ART and the last CD4 count at the time of stability group assignment. Stability group was assessed as of 31 December 2014. Outcomes were defined as retention in care, death, lost to follow up (>60 days since the last appointment) and treatment failure (clinical failure as occurrence of the new WHO stage III or IV clinical condition and immunological failure as drop in CD4 by 30% or more). Outcomes were assessed one year after stability group assignment on 31 December 2015, and reported as rates. Calculation for the total number of physician-averted visits in 2015, was done by taking into account number of averted visits per year for group B (2 averted visits/patient/year) and group C (4 averted visits/patient/year) multiplied by the number of patients in group B and C. Before the implementation of the differentiated model of care physicians consulted all patients on each clinical visit (minimum 4 times/patient/year). With the new model of care B patients see a physician only twice per year (2 averted visits/patient/year) while group C patients do not have routinely scheduled physician’s consultations (4 averted visits/patient/year).

## Results

As of 31 December 2014, MSF’s Yangon clinics were providing HIV care to 16, 272 adult patients, including 167 receiving comprehensive DRTB care in 2015. Data on non-communicable diseases were not available in 2015; however, data from 2016 revealed that some 10% of the Yangon cohort received treatment for hypertension and 2% for diabetes mellitus. The stability group accounted for 15, 629 (96%) of adult patients, and according to established criteria, 12, 557 (80%) were considered as clinically stable; 8, 001 (51.19%) as group C and 4, 556 (29.15%) as group B. The age and gender distribution within groups were similar, while the duration of care and last CD4 count distribution were mostly consistent with the stability criteria (Table 2). 34 patients were classified as group C with ART duration <12 months, which is considered as data misclassification.

**Table 2.** Characteristics of adult patients on 31st December 2014.

	Group A	Group B	Group C
Variable	*N* (%)	*N* (%)	*N* (%)
Total^a^	3, 072 (19.66%)	4, 556 (29.15%)	8, 001 (51.19%)
Gender			
Male	1, 692 (55.08%)	2, 729 (59.90%)	4, 432 (55.39%)
Female	1, 380 (44.92%)	1, 827 (40.10%)	3, 569 (44.61%)
Age (years)			
18–45	2, 442 (79.49%)	3, 461 (75.97%)	6, 480 (80.99%)
>45	630 (20.51%)	1, 095 (24.03%)	1, 521 (19.01%)
Time on ART at time of enrolment into	159 (5.18%)	23 (0.50%)	5 (0.06%)
ABC group (months):	235 (7.65%)	378 (8.30%)	29 (0.36%)
<6 months	842 (27.41%)	1, 105 (24.25%)	975 (12.19%)
6 to <12 months	1, 836 (59.77%)	3, 050 (66.94%)	6, 992 (87.39%)
12 to <24 months			
≥24 months			
CD4 count^b^	2, 307 (75.10%)	3, 921 (86.09%)	7, 715 (96.43%)
≥200 cells/mm^3^ <200 cells/mm^3^ Not available	760 (24.74%) 5 (0.16%)	629 (13.81%) 6 (0.13%)	286 (3.57%)-

^a^ Under care = not deceased, not transferred out and who had an appointment after December 2014.

^b^ Last CD4 count available by December 2014.

In 2012, 20 medical teams were needed to provide care to 13, 705 patients. By comparison, just 10 medical teams were needed to provide care for 16, 272 patients in 2014 (Table 3). Furthermore, assuming that stable patients do not change status during the 12-month period, we estimate that in 2015 alone, the new model averted 41, 116 physician visits (4, 556 B patients as of 31 December 2014 × 2 physician visits and 8,001 C patients as of December 2014 × 4 physician visits), as patients could instead be seen by a nurse or refill their ARTs at the pharmacy.

**Table 3.** Average number of adult patients under care per medical team (2012–2015).

Year	Number of patients under care	Number of medical teams^a^ in the project	Average number of patients under one team
Dec 31 2011	11, 168	15	745
Dec 31 2012	13, 705	20	685
Dec 31 2013	16, 436	17	967
Dec 31 2014	16, 272	10	1, 627

^a^A team that comprises a physician, a nurse and a counsellor. The number of teams is based on the number of physicians (1 physician = 1 team).

Table 4 presents treatment outcomes one year after implementation of the model for stable adult patients on ART. Rates of retention in care were measured as 97.78% and 98.81% for group B and C respectively. During the year 51 stable patients died, unfortunately the cause of death could not be determined for the purpose of this analysis. LFU rates were low, reaching 1.21% for group B and 0.64% for group C. Rates of immunological failure for group B and C were similar, 6.01% and 5.72%, respectively. During the year period, most of the patients remained within the same group as assigned in the previous year. However 666 (14.60%) group B and 656 (8.20%) group C patients moved back to group A, due to either to treatment failure, clinical deterioration related or unrelated to HIV or drug side effects.

**Table 4.** One year treatment outcomes (31st Dec 2015) for adult patients under care who were in B or C treatment groups on 31st Dec 2014.

	Group B (n = 4, 556) N (%)	Group C (n = 8, 001) N (%)
Remained under care	4, 455 (97.78%)	7, 906 (98.81%)
Dead	32 (0.70%)	19 (0.24%)
Lost to follow up^a^	55 (1.21%)	51 (0.64%)
Transferred out^b^	14 (0.31 %)	25 (0.31%)
New WHO clinical stage 3 or 4	37 (0.81%)	29 (0.36%)
Drop in CD4 count by 30%^c^	274 (6.01%)	458 (5.72%)
Transferred back to group A	666 (14.60%)	656 (8.20%)

^a^Data extracted as of 31 December 2015, therefore anyone with next appointment date <1 November 2015 (60 days) is considered LTF.

^b^Transferred out from MSF to the National AIDS Programme or other NGO.

^c^CD4 count result as of December 2014 was only available for 12, 551 (99.95%) B and G group patients.

## Discussion

Our study is the first one describing differentiated HIV care in Myanmar and our model has shown good patient outcomes for patients stable on ART. Implemented model is the facility based and includes differentiation by age, duration of ART and clinical characteristics as well as task shifting, reduction of frequency of clinical visits with separation of drug refills. The majority of our patients on ART (80.34%) were defined as stable. One-year treatment outcomes were good with aggregated rates of retention in care for stable patients of 98.4% and low rates of death (0.4%), LFU (0.9%) and clinical (0.8%) or immunological failure (5.8%). Similar outcomes are reported after implementation of differentiated care in different resource limited contexts [17]. In 2012, 20 medical teams were needed to provide care to 13,705 patients. By comparison, just 10 medical teams were needed to provide care for 16,272 patients in 2014, suggesting that introduction of the new model has made our service delivery more efficient, allowing more patients to be followed by smaller number of staff. Together, task shifting and reduction of intensity of follow up achieved saving of large number of physicians’ consultations (41, 116 physician visits averted in 2014), creating extra physicians’ time for co-morbid, severely sick or patients failing ART. Research conducted by Alamo et al. presents evidence that such approaches improve patient flow, reduce waiting time and increase patients’ and providers’ satisfaction [10]. At the same time, studies conducted in similar urban context of Uganda show that facility-based interventions are cost-effective and have positive effect on patient’s adherence [11,12]. During the first year of intervention, majority of stable patients remained in the same stability group with only 1322 (10.53%) returning back to group A. Treatment failure remains one of the main reasons for transfer to unstable group, and this urges us to keep emphasizing adherence support mechanisms even for stable patients, as well as to increase access to routine viral load for this group. Improved access to viral load monitoring will enable further simplification of care; patients will be able to enter stable groups six months after initiation of ART, if undetectable [18,19].

The initial set up of fixed medical teams responsible for a defined cohort of patients has been kept with ambition to increase size of the cohort under the responsibly of one physician. In our experience, fixed medical teams can successfully implement task sharing between nurses and physicians, while respecting Myanmar national regulations for nurse responsibilities (e.g. nurses are not responsible for ART prescriptions) [20]. This results in better supervision of the nurses new to clinical consultations, and in positive patient–provider relationships, a key factor in achieving retention in care [21–23]. At the same time such model would reflect a real situation in the public health sector with limited number of physicians in decentralized sites.

The National AIDS Programme emphasizes importance of transitioning of the HIV service delivery from centralized and specialized to decentralized, differentiated and based on task shifting models of care [20]. Decentralization of HIV care in higher prevalence areas is planned down to the level of rural health centres, where only basic health staff is available [14,20]. Task shifting to support the scale up of HIV by the NAP would require systematic training and empowerment of nurses and basic health staff involved in HIV care at the national level, as is already underway in other contexts [24]. We believe that with such ambitions, similar to ours, models of care can be implemented in the public sector.

There are several limitations to this study. First, this is a retrospective analysis and it was conducted just one year after implementation of the model was completed. This is too short a period to evaluate treatment outcomes over time. Nevertheless, this initial analysis suggests that the model is effective. Second, we were not able to report virological outcomes in our cohort, due to limited access to routine viral load at the time of analysis. Information about virological suppression among patients differentiated to ART refills only (long term stable groups C), will help us in the future to access more accurately if this simplified model of care influences ART treatment response. Furthermore, the study did not investigate patient’s perspectives on the model, or attempt to assess their level of satisfaction, as is recommended for any comprehensive analysis of patient-centred services [25]. A satisfaction survey should be performed and service delivery should be adapted accordingly. Last, notably lacking in our model is the community involvement in ART delivery and analysis of its specific role in the context of an urban, concentrated HIV epidemic in Myanmar, similar to what has been done in contexts of generalized HIV epidemic in Sub-Saharan Africa [26,27].

## Conclusions

Described model of differentiated HIV care adapted to the Myanmar context reduced the number of visits between stable patients and physicians, reduced the medical resources required for maintaining the stable HIV cohort on ART and enabled integrated treatment of the main co-morbidities. As Myanmar’s NAP strives to attain the 90-90-90 global fast track targets set by UNAIDS and national guidelines evolve toward initiation of ART for all patients, differentiation of care is critical to ensuring the quality of HIV services and alleviating the burden of the epidemic on the health system. The MSF model of differentiated care presents a first step on the path to simplification of HIV care and being based on limited human resources it can be replicated in less urban settings of Myanmar. At the same time, we believe that further adaptation of this model is possible, and that future iterations emphasize community involvement. We hope that this example demonstrates to stakeholders the immense potential of differentiation of HIV care to accelerate the national scale up of ART, the decentralization of services and the integration of care for the main co-morbidities in Myanmar.

## References

[1] National AIDS Programme Myanmar National HIV-AIDS Strategic Plan 2016-2020. Naypyidaw, Myanmar: Ministry of Health and Sports; 2016 Sep.

[2] National AIDS Programme Myanmar Global AIDS Response Progress Report Myanmar. Naypyidaw, Myanmar: Ministry of Health and Sports; 2015 6.

[3] World Health Organisation Global Tuberculosis Report. Naypyidaw, Myanmar: Ministry of Health and Sports; 2015.

[4] World Health Organisation Atlas of Substance Use Disorders. Country Profile Myanmar. 2010 Available from: http://www.who.int/substance_abuse/publications/atlas_report/profiles/myanmar.pdf

[5] Zaw SKK, Tun STT, Thida A, Aung TK, Maung W, Shwe M, et al Prevalence of hepatitis C and B virus among patients infected with HIV: a cross-sectional analysis of a large HIV care programme in Myanmar. Trop Doct. 2013 7;43(3):113–13. 23800421 10.1177/0049475513493416

[6] Minstry of Health Myanmar Simplified Treatment Guideline for Hepatitis C Infection. Naypidaw, Myanmar: Ministry of Health and Sports; 2015.

[7] Department of Public Health Ministry of Health Myanmar Myanmar STEPS Report. Naypidaw, Myanmar: Ministry of Health and Sports Myanmar; 2016.

[8] Ministry of Immigration and Population Myanmar The 2014 Myanmar Population and Housing Census. Naypidaw, Myanmar: Ministry of Health and Sports; 2015.

[9] Sabapathy K, Ford N, Chan KN, Kyaw MK, Elema R, Smithuis F, et al Treatment outcomes from the largest antiretroviral treatment program in Myanmar (Burma): a cohort analysis of retention after scale-up. J Acquir Immune Defic Syndr. 2012 6;60(2):e53–62. 22334069 10.1097/QAI.0b013e31824d5689

[10] Alamo ST, Wagner GJ, Ouma J, Sunday P, Marie L, Colebunders R, et al Strategies for Optimizing Clinic Efficiency in a Community-Based Antiretroviral Treatment Programme in Uganda. AIDS Behav. 2013 5 20;17(1):274–83. 22610422 10.1007/s10461-012-0199-9 PMC3887144

[11] Obua C, Kayiwa J, Waako P, Tomson G, Balidawa H, Chalker J, et al Improving adherence to antiretroviral treatment in Uganda with a low-resource facility-based intervention. Global Health Action. 2014 11 20;7(1):24198–9. 10.3402/gha.v7.24198 PMC4049133 24909408

[12] Babigumira JB, Castelnuovo B, Stergachis A, Kiragga A, Shaefer P, Lamorde M, et al Cost Effectiveness of a Pharmacy-Only Refill Program in a Large Urban HIV/AIDS Clinic in Uganda. van Baal P, editor Plos One. 2011 3 28;6(3):e18193–7. 21464895 10.1371/journal.pone.0018193 PMC3065481

[13] World Health Organisation Consolidated Guidelines on The Use of Antiretroviral Drugs for Treating and Preventing HIV Infection. Geneva: WHO; 2016 Jun. 27466667

[14] National AIDS Programme Myanmar Interim Operational Guidance for HIV Service Delivery in Myanmar. Naypidaw, Myanmar: Ministry of Health and Sports; 2017.

[15] Medecins Sans Frontieres MSF Ethics Review Board Standard Operating Procedures. Geneva; 2013 p. 1–1. Available from: http://fieldresearch.msf.org/msf/bitstream/10144/294968/5/MSF+ERB+SOP+Dec+2013.pdf

[16] National AIDS Programme Guidelines for the Clinical Management of HIV Infection in Myanmar. Naypidaw, Myanmar: Ministry of Health and Sports; 2015 Jun.

[17] Bemelmans M, Baert S, Goemaere E, Wilkinson L, Vandendyck M, Van CUTSEM G, et al Community-supported models of care for people on HIV treatment in sub-Saharan Africa. Trop Med Int Health. 2014 5 28;19(8):968–77. 24889337 10.1111/tmi.12332

[18] Phillips A, Shroufi A, Vojnov L, Cohn J, Roberts T, Ellman T, et al Sustainable HIV Treatment in Africa through Viral Load-Informed Differentiated Care. Nature. 2015 12 3;528(7580):S68–S76. 26633768 10.1038/nature16046 PMC4932825

[19] Bonner K, Mezochow A, Roberts T, Ford N, Cohn J. Viral load monitoring as a tool to reinforce adherence: a systematic review. J Acquir Immune Defic Syndr. 2013 9;64(1):74–8. 23774877 10.1097/QAI.0b013e31829f05ac

[20] National AIDS Programme Myanmar Standard Operating Procedures (SOP) for the Decentralized Site for ART Services in Myanmar. Naypidaw, Myanmar: Ministry of Health and Sports; 2015.

[21] Callaghan M, Ford N, Schneider H A systematic review of task- shifting for HIV treatment and care in Africa. Hum Resour Health. 2010;8(8):1–9. 20356363 10.1186/1478-4491-8-8 PMC2873343

[22] Yehia BR, Schranz AJ, Momplaisir F, Keller SC, Gross R, Frank I, et al Outcomes of HIV-Infected Patients Receiving Care at Multiple Clinics. AIDS Behav. 2014 8 1;18(8):1511–22. 24077931 10.1007/s10461-013-0625-7 PMC3969411

[23] Fox MP, Ive P, Long L, Maskew M, Sanne I High rates of survival, immune reconstitution, and virologic suppression on second-line antiretroviral therapy in South Africa. J Acquir Immune Defic Syndr. 2010 4;53(4):500–6. 19838128 10.1097/QAI.0b013e3181bcdac1

[24] Middleton L, Howard AA, Dohrn J, Zinkernagel Von D, Parham Hopson D, Aranda-Naranjo B, et al The Nursing Education Partnership Initiative (NEPI). Acad Med. 2014 8;89(Supplement):S24–8. 25072571 10.1097/ACM.0000000000000342

[25] The Global Fund A toolkit for health facilities. Differentiated Care For HIV and Tuberculosis. Geneva; The Global Fund; 2015 Dec.

[26] Médecins Sans Frontierès Community ART Group Toolkit. Brussels; 2013 Dec.

[27] Médecins Sans Frontierès Reaching Closer to Home. Brussels: Médecins Sans Frontierès; 2013 Nov.

